# Chronological dynamics of the gut microbiome in response to the pasture grazing system in geese

**DOI:** 10.1128/spectrum.04188-23

**Published:** 2024-08-27

**Authors:** Qasim Ali, Sen Ma, Umar Farooq, Boshuai Liu, Zhichang Wang, Hao Sun, Yalei Cui, Defeng Li, Yinghua Shi

**Affiliations:** 1 Department of Animal Nutrition and Feed Science, College of Animal Science and Technology, Henan Agricultural University, Zhengzhou, China; 2 Henan Key Laboratory of Innovation and Utilization of Grassland Resources, Zhengzhou, China; 3 Henan Herbage Engineering Technology Research Center, Zhengzhou, China; 4 Department of Poultry Science, University of Agriculture Faisalabad, Sub Campus Toba Tek Singh, Toba Tek Singh, Pakistan; Nanjing Agricultural University, Nanjing, China

**Keywords:** pasture grazing system, geese, gut microbiota, short-chain fatty acids, metabolic functions

## Abstract

It is commonly accepted that dietary fibers are good for gut health. The effect of fibers on the diversity and metabolic activities of the cecal microflora, however, differ with the passage of time. Therefore, we investigated the time-series impacts of the pasture grazing system (a high dietary fiber source) on the cecal microbiome and short-chain fatty acids in Wanpu geese, comparing it to commercial feeding (a low dietary fiber source). The cecal microbiota composition and SCFA concentrations were evaluated by 16S rRNA gene sequencing and gas chromatography, respectively. We found that pasture produced a generally quick positive response to Bacteroidales, Lactobacillales, Gastranaerophilales (at 45 days), Lachnospirales, and Oscillospirales (at 60 days and 90 days) irrespective of Erysipelotrichales (at 45 days), *Clostridia_UCG-014*, *RF39* (at 60 days), Christensenellales, and Peptostreptococcales-Tissierellales (at 90 days) in geese. Meanwhile, we found that Lactobacillales, Gastranaerophilales, Lachnospirales, and Oscillospirales were significantly correlated with short-chain fatty acids in pasture grazing geese. Indeed, the correlation of cecal microbiota with SCFAs led to altered microbial functions evinced by COG; KEGG pathway levels 1, 2, and 3; BugBase; and FAPROTAX databases. This study emphasizes the importance of dietary fiber sources in influencing beneficial impacts in regulating geese microbiota homeostasis and metabolic functions such as energy and lipid metabolism.

**IMPORTANCE:**

Low dietary fiber diet sources cause gut microbial and short-chain fatty acid alterations that lead to compromised animal health. The establishment of an artificial pasture grazing system at the expense of ryegrass is a good source of dietary fiber for geese. Our results described the importance of pasture in maintaining the gut microbiota, SCFAs, and potential microbial functions reported by COG; KEGG pathway levels 1, 2, and 3; BugBase; and FAPROTAX databases.

## INTRODUCTION

With a fast-growing population, the demand for high-quality poultry meat is increasing worldwide (1). The continuous use of antibiotics has not only changed the gut microbiota in poultry but also threatened human health by influencing antibiotic resistance genes (ARGs) (2). Therefore, a globe-wide search for alternatives has been triggered over time. In this regard, dietary fiber has gained attention in influencing the gut microbiota. During the past 10 years, the role of the microbiome has been intensively highlighted in the areas of health and disease in humans and animals (3, 4). Studies have revealed the critical role of gut microbiota in nutrient absorption (5), development of host (1), diseases (6), immunity (7), synthesis of vitamin, bile acids, and metabolism of sterols (8).

**Fig 1:**
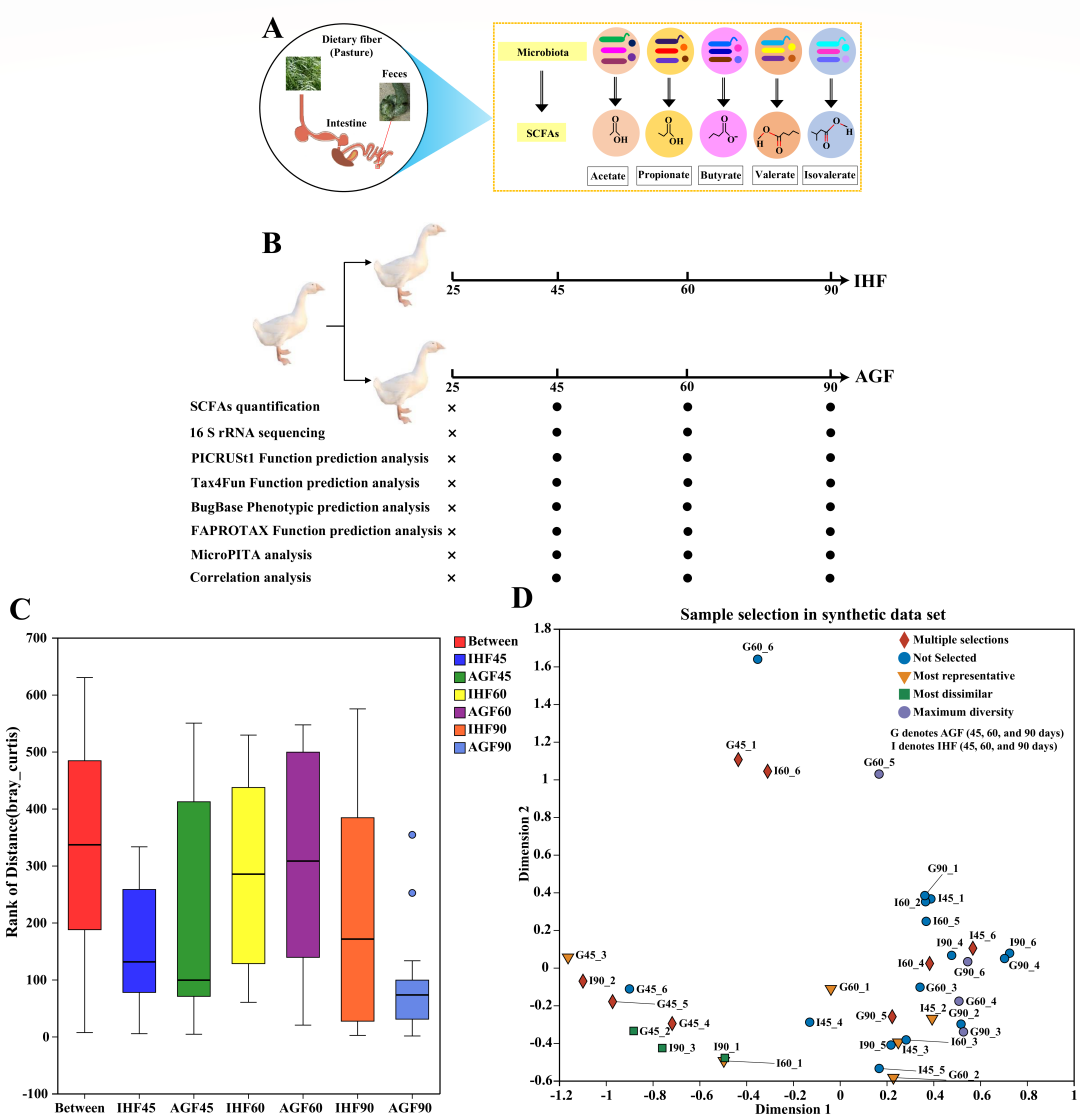
Longitudinal profiling of geese gut microbiota and metabolites to study the commercial diet-dependent modulations in response to pasture intervention. (**A**) An ecological perspective of diet-dependent dynamical responses of gut microbiota and SCFAs. Supplementation of pasture alters the composition of gut microbiota. Among them, a few bacteria play a key metabolic role as SCFA fermenters. (**B**) Experimental design: 90 geese from the first day of life were fed a commercial diet, and 90 geese from 25 days of age were fed with a commercial diet (once in a day, 19:00 p.m.) and pasture (6:00 a.m. to 18:00 p.m.). Black bolts indicate the days on which the data were collected from the cecal chyme. (**C**) ANOSIM-Adonis analysis (Bray–Curtis) was developed to find significant differences among the groups. (**D**) microPITA was used to screen representative samples from a large number of microbial diversity samples according to different criteria for metagenomic sequencing.

The gut microbiota in geese is influenced by diet (9), host genetics (10), and environmental factors (11). However, few reports have been previously reported on the role of diet (particularly dietary fiber) in modulating the gut microbiota (12, 13). Dietary fibers consist of non-starch polysaccharides and non-carbohydrate components that are only degraded by the gut microbiota, mainly found in the cecum and colon. In the large intestine (particularly the cecum), the end products of microbial fermentative activities are short-chain fatty acids (SCFAs), which improve animal health (14) (Fig. 1A). The lowered abundance of SCFAs has been associated with obesity, inflammatory bowel disease (IBD), and autoimmune disorders (15–17). The introduction of dietary fibers as prebiotics has been intended to restore gut microbiota by influencing SCFA concentrations in broiler chickens (18).

Previous studies revealed that dietary fibers affect the composition of microflora (17, 19). The ability of the fibers to influence SCFA synthesis differs among different individuals (20, 21). Mohan Qiu *et al*. revealed that alfalfa meal promotes the production of SCFAs only when used at rates of 5%, 7%, or 9% in geese (22). Similarly, in another study, the use of alfalfa meal and peanut vine as dietary fiber sources was observed to induce butyric acid in rabbits (23). Individual-to-individual variation in metabolomics and bacterial features of the intestinal microbiome (12, 24) can additionally improve biological dynamics such as energy metabolism (22, 25) and lipid metabolism (26).

In this study, we investigated the chronological response of cecal microbiota in response to the pasture grazing system in Wanpu geese. We found a time-series response of key cecal microbiota to SCFA production in response to the pasture grazing system. We further show that the cecal microbial composition predicts the individualized response of SCFAs to COG functional features and KEGG pathways at levels 1, 2, and 3. Indeed, the crosstalk of SCFAs with these functional features and pathways deciphered the energy and lipid metabolism in geese.

## RESULTS

### Wanpu geese from the different feeding systems vary in their gut microbiota composition

The Wanpu geese anchorage microbiota can be used to study nutritional interventions. Prior to fiber interventions, all geese were fed with a commercial starter diet till 25 days. We determined chronological changes in microflora, SCFA concentration (by targeted metabolomics), and phenotypic shifts following exposure to pasture (ryegrass, a fiber source) and commercial diet (Fig. 1B). The pasture used in this study was able to support gut microbiota in the cecum (13) and regulate the synthesis of SCFAs (27).

The ANOSIM analysis explains the significant differences between the groups or within the groups, while the Adonis analysis uses a distance matrix to decompose the total variance. In our study, we used ANOSIM-Adonis analysis to determine a significant difference among the groups. In ANOSIM (Bray–Curtis), the calculated *R-value* for distance among groups on the order level was 0.2947, and the *P-value* was 0.001. Herein, the *R-value* is less than 1, indicating no significant difference among the groups, whereas, for Adonis (Bray–Curtis), the *R-value* for group factors (5) was 0.36, and for residual (sampling, 30), it was 0.64, describing that the interpretation degree of the grouping to the difference was less as compared to the interpretation degree of the sampling to the difference (Fig. 1C; Table S1).

Then, to exemplify the five criteria for microbial community selection, we used microPITA to generate a number of synthetic communities in a stage study design (Fig. 1D; Table S2). Each group consisted of six samples; in total, 36 synthetic taxa were generated. Among these samples, one group (AGF) exhibited maximum diversity (with round light blue blocks) at 45 days (3), 60 days (3), and 90 days (4). Two groups were specified for differential abundance in blocks of four or six taxa to create dissimilarity among themselves (four samples of AGF at 45 days, one sample of IHF at 45 days, two samples of IHF at 60 days, and three samples of IHF at 90 days, with green squares exhibiting the most dissimilarity). Furthermore, two groups were gifted for higher abundance in blocks of four or six taxa to make greater presentation among themselves (one sample of AGF at 45 days, two samples of AGF at 60 days, one sample of AGF at 90 days, three samples of IHF at 45 days, two samples of IHF at 60 days, and one sample of IHF at 90 days with gold triangles exhibiting the most representative).

### Commercial diet-dependent gut microbiota dynamics in response to pasture interventions

**Fig 2:**
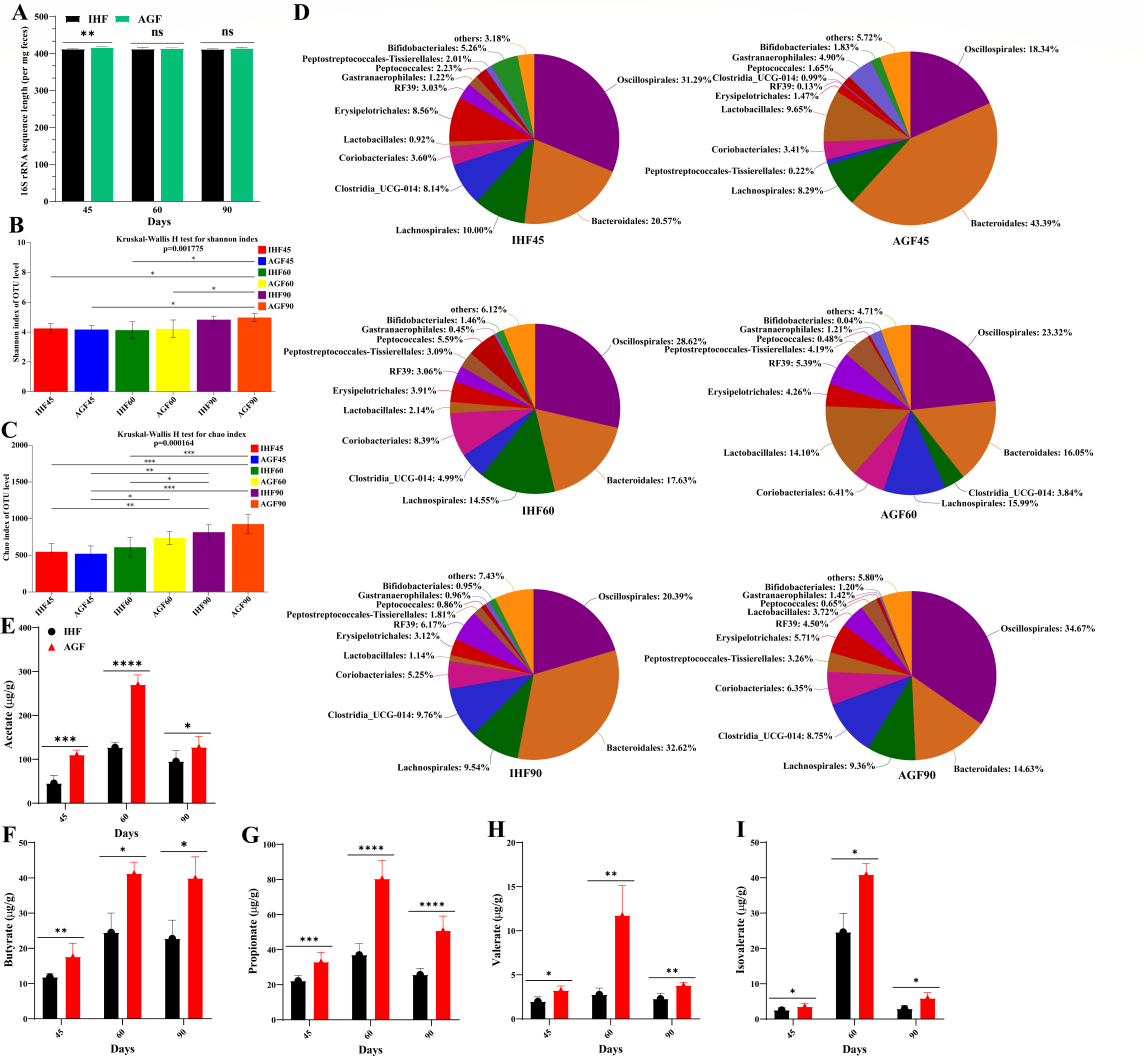
Pasture-induced temporal shifts in the geese cecal microbiome and short-chain fatty acid (SCFA) metabolism. (**A**) Bacterial load. (**B and C**) Alpha diversity of cecal microbiota composition (Shannon and Chao index of OTU level). (**D**) Relative abundance of cecal microbiota at the order level shown in a pieplot. (**E-I**) Fecal concentration of acetate, butyrate, propionate, valerate, and isovalerate. In-house feeding system (IHF) and artificial pasture grazing system (AGF). Data with different superscript letters are significantly different (*P* < 0.05) according to the unpaired Student’s *t*-test. The asterisk symbol indicates significant differences *P < 0.05, **P < 0.01, and ***P < 0.001.

Pasture feeding suddenly increased the concentration of cecal microbiota on a time-scale basis (Fig. 2A). Initially, the pasture dropped the cecal microbiota diversity at 45 days and then restored it (Fig. 2B and C). In fact, we identified quick but non-monotonic alterations in the relative abundances of dominant bacteria at order levels such as Oscillospirales and Bacteroidales (Fig. 2D). Notably, Oscillospirales gradually increased in AGF geese at 45 (18.34%), 60 (23.32%), and 90 days (34.67%), and Bacteroidales tentatively decreased from 45 to 90 days (i.e., 45 days; 43.39%, 60 days; 16.05%, and 90 days; 14.63%). A meta-analysis of five microbiota studies demonstrated that the decrease in Oscillospirales is concomitantly correlated with IBD (28) and pediatric nonalcoholic steatohepatitis (29) in IHF geese. The spread of ARGs through antibiotic treatment for bacterial infection leads to a growing threat to human health (30). ARG-bearing bacteria can transfer from the environment to humans and then possess a negative effect on human health (31). In a recent study, Bacteroidales species have been honored for regulating ARGs in humans (32). According to the above studies, the non-monotonic but rapid increase of Bacteroidales in IHF geese could be an alarming condition that may directly impact human health.

The cecal microflora alteration was escorted by alterations in the concentrations of three main SCFAs (acetate, butyrate, and propionate), valerate, and isovalerate (Fig. 2E through I). Since bacterial fermentation of the pasture produces these metabolites, we assume similar time-dependent dynamics of SCFA concentrations. In fact, both total (acetate, butyrate, propionate, valerate, and isovalerate) and individual SCFAs show two chronological phases: their concentrations spiked in the short term (at 60 days) before slowly decreasing until steady states, with an exemption of AGF geese (90 days) whose butyrate level was notably constant. The main concentration ratios of total SCFAs were 17.25, 33.69, 43.66, 89.53, 29.24, and 45.82 for IHF45, AGF45, IHF60, AGF60, IHF90, and AGF90 groups, respectively (Table S3). In addition, the total SCFA concentration for all groups at 45, 60, and 90 days was 25.47, 66.59, and 37.53, respectively.

**Fig 3:**
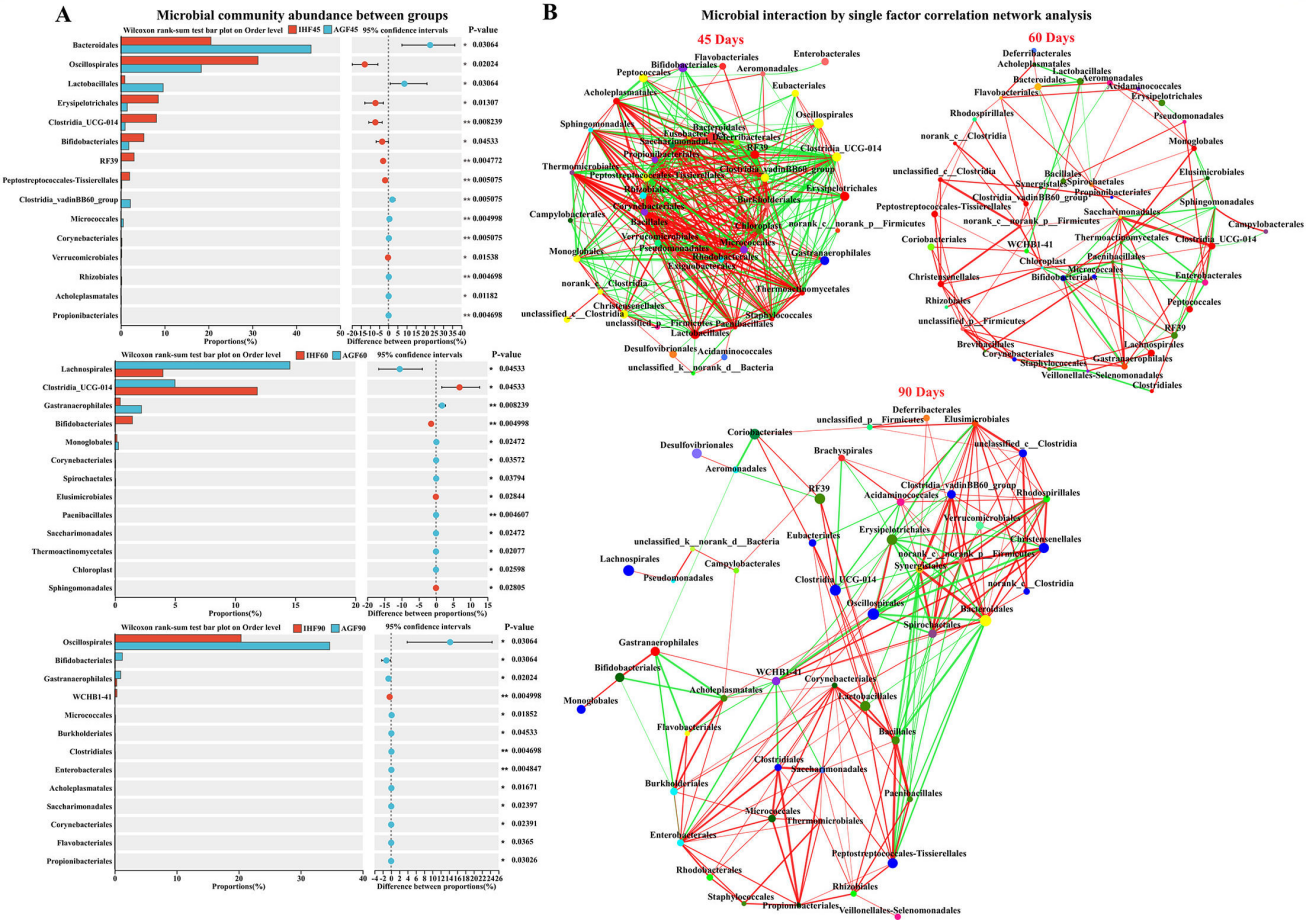
Commercial diet-dependent gut microbiota dynamics in response to pasture interventions. (**A**) Wilcoxon rank-sum test for two sets of independent samples. (**B**) Single-factor correlation network analysis to reflect species correlation. Each co-occurring pair among host markers had an absolute Spearman’s correlation above 0.50 [the red thin line indicates a general positive correlation (0.5 < R < .70); the red thick line indicates a strong positive correlation (R > 0.70); the green thin line indicates a general negative correlation (−0.7  <  R < −0.50)] with a significance level under 0.05. Circles with distinct colors represent the species from the order level. Edge lines with distinct colors are in direct proportion to the direction of the correlation (red means positive correlation; green means negative correlation). The width of the edge line represents the strength of the correlation.

Next, based on the community abundance data in the sample, the Wilcoxon rank-sum test, also known as the Mann–Whitney U test, was applied for testing two sets of independent samples (Fig. 3A). Bacteroidales and Lactobacillales were significantly highly abundant in AGF geese compared with IHF geese at 45 days of age. Similarly, Lachnospirales and Gastranaerophilales were increased in AGF geese at 60 days compared with those of IHF geese. At 90 days of age, Oscillospirales, Bifidobacteriales, and Gastranaerophilales were augmented in AGF geese than in the IHF geese. After confirming the community difference between the two groups, we applied a single-factor correlation network analysis to reflect species correlation at the order level under a certain environmental condition. The top 50 species in total abundance at a taxonomic level were selected, and correlation coefficients such as Spearman rank between species were calculated to reflect the correlation between species at 45, 60, and 90 days (Fig. 3B). The size of the nodes in the figure indicates the abundance of species. The red line indicates a positive correlation, and the green line indicates a negative correlation. The thickness of the line indicates the size of the correlation coefficient, and the thicker line represents the higher correlation between species.

### Fit to neutral model of community assembly

**Fig 4:**
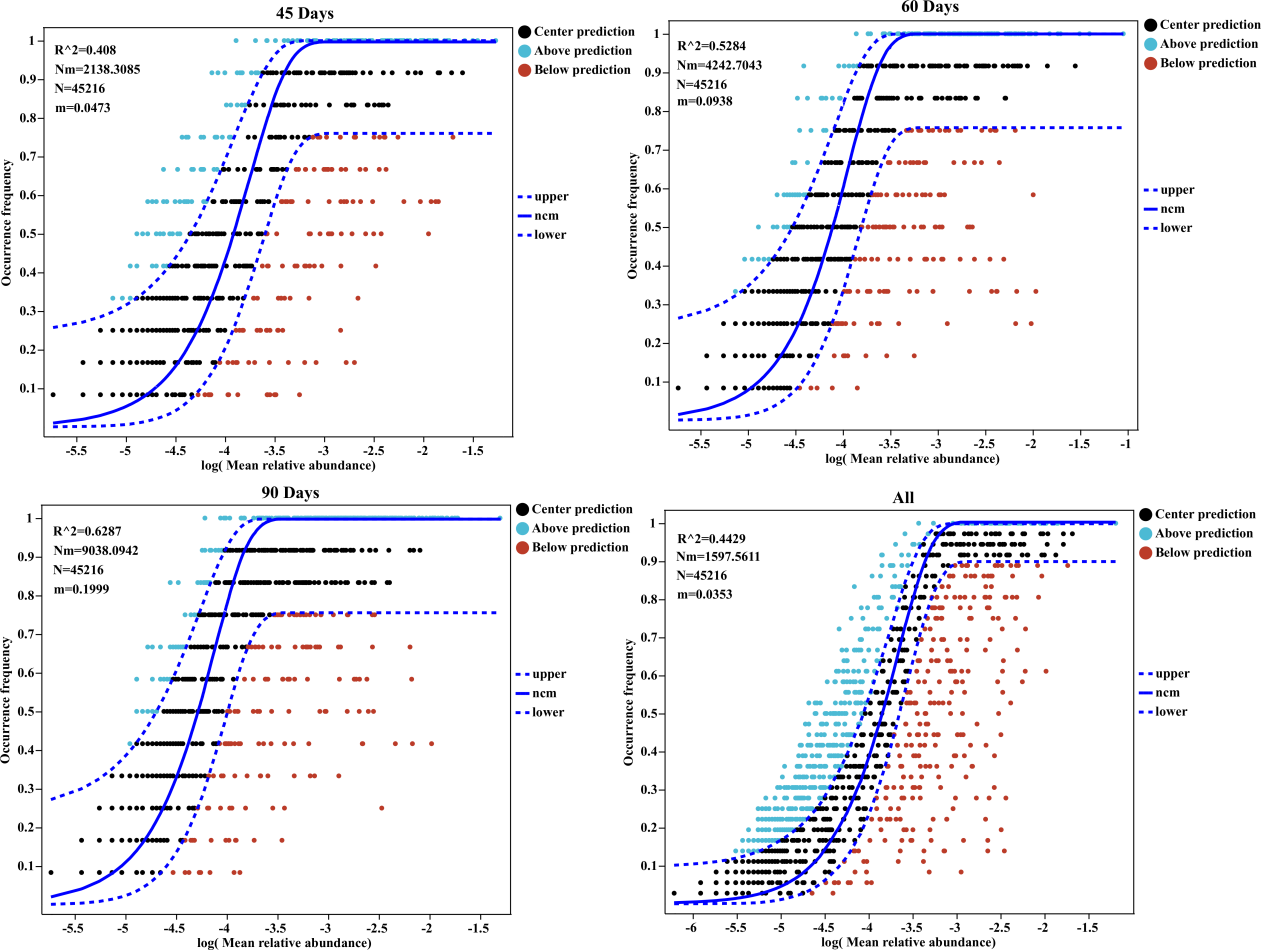
Fit to neutral model of community assembly. The predicted occurrence frequencies for 45, 60, 90, and all days of sample examination represent the occurrence frequency of genera and their relative abundance variations in the cecal chyme of geese. The solid line represents the fit of the neutral model, and the dashed upper and lower lines represent the 95% confidence level of the model’s predictions, N describes the metacommunity size and the total abundance of all genera in each sample, m quantifies the migration rate at the community level, *Nm* the product of meta-community size (**N**) and mobility (**M**) (*Nm* = N*m) quantifies the estimation of diffusion between communities and determines the correlation between frequency of occurrence and regional relative abundance, and R^2^ represents the overall goodness-of-fit of the neutral community model.

The neutral community model (NCM) has been shown to describe the importance of the observed species abundance distribution in large biomes. Herein, we identified the relationship between the occurrence frequency of genera and their relative abundance variations (Fig. 4), with 40.80%, 52.84%, 62.87%, and 44.29% of explained community variance for 45, 60, 90, and all days of sample examination, respectively. The R^2^ represents the overall goodness of fit of the neutral community model. A lower R^2^ value for 45 days (0.408) and all days of sample examination (0.4429) was observed in this study, indicating that it is not close to the neutral model. We determined higher R^2^ values for 60 days (0.53) and 90 days (0.63), demonstrating that it is almost close to the neutral model. The *Nm*-value was higher for microbial communities at 90 days (*Nm* = 9038.09) followed by 60 days (*Nm* = 4242.70), 45 days (*Nm* = 2138.31), and all days of sample examination (*Nm* = 1597.56). Since the number of sequences in both samples was 45,216, the estimated m value for 45, 60, 90, and all days of sample examination was 0.0473, 0.0938, 0.1999, and 0.0353, respectively. These results represented that species dispersal was higher in the 90-day-old geese than in 60-day-old and 45-day-old geese.

### Functional features of the gut microbiota isolated from Wanpu geese

**Fig 5:**
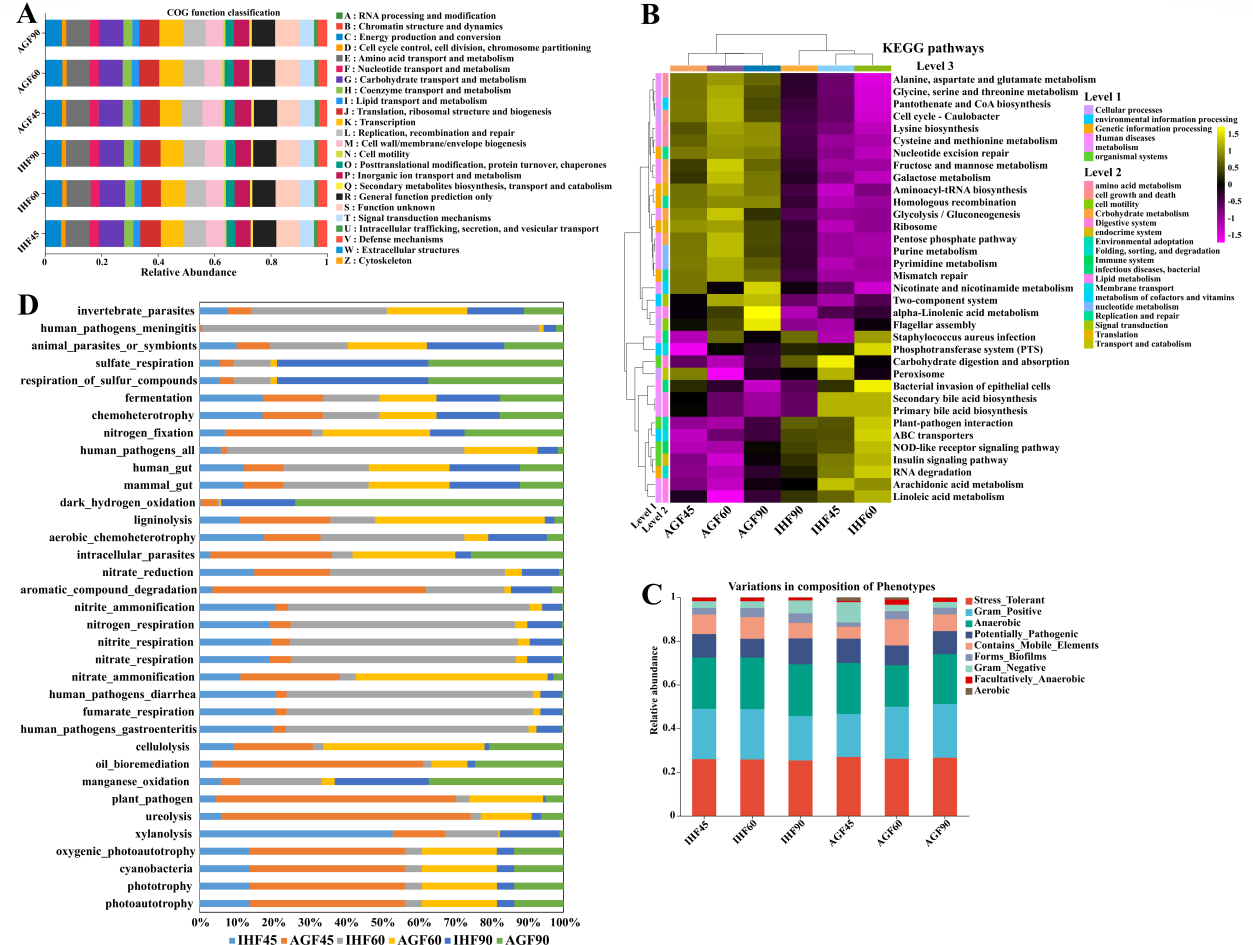
Functional features of the gut microbiota isolated from Wanpu geese. (**A**) The microbial functional features in Wanpu geese were measured by PICRUSt1 software to analyze the data in the context of the COG database. (**B**) Functional predictions of the gut microbiota between the IHF and AGF geese groups generated from 16S rRNA gene sequences using Tax4Fun software were performed under a clustering heatmap with default parameters. The small square represents a KEGG pathway at level 3, its color represents the amount of KEGG pathway expression, yellow represents a high-expression KEGG pathway, and purple represents a low-expression KEGG pathway. The treemap at the top of Fig. B represents the clustering results of different samples from different experimental groups at 45, 60, and 90 days within their respective results of KEGG pathways at level 3, and the treemap on the left represents the clustering results of different KEGG pathways at levels 1 and 2 from different samples. (**C**) BugBase phenotypic prediction. (**D**) FAPROTAX functional prediction.

To comprehend how the microbiota plays an important role in meat geese at 45, 60, and 90 days, the PICRUSt1 (phylogenetic investigation of communities by reconstruction of unobserved states) program was applied to analyze the data in the context of the Cluster of Orthologous Groups (COG). By using this program, we found microbial functional features based on COG functional classification (Fig. 5A). The functional features include RNA processing and modification, chromatin structure and dynamics, energy production and conversion, cell cycle control, cell division, chromosome partitioning, amino acid transport and metabolism, nucleotide transport and metabolism, carbohydrate transport and metabolism, and coenzyme transport and metabolism that were increased at 45 days, then suddenly decreased at 60 days, and finally maintained at a steady state (90 days). The lipid transport and metabolism tended to increase at 45 days, but further decreased at 60 days and 90 days in AGF geese compared to IHF geese.

Further, the functional alterations of gut microbial KEGG were predicted using the Tax4Fun between the two groups at 45, 60, and 90 days (Fig. 5B). Predominant KEGG pathways at level 1 were cellular processes, environmental information processing, genetic information processing, human diseases, metabolism, and organismal systems (Fig. S1). Of the 41 KEGG pathways recovered at level 2 in the IHF and AGF geese groups, 18 showed altered expression (Fig. S1) and 23 showed under-expression (data not shown). Most of the predominant KEGG pathways were involved in metabolism (i.e., carbohydrate metabolism, metabolism of cofactors and vitamins, nucleotide metabolism, lipid metabolism, and amino acid metabolism) and organismal systems (i.e., endocrine system, immune system, environmental adaptation, and digestive system) (Fig. S1). Furthermore, 257 KEGG pathways were identified at level 3 in IHF and AGF geese. Among them, we identified the highly abundant differentially significant (*P* < 0.05) 35 pathways on KEGG level 3 (Fig. S1).

BugBase makes functional predictions on bacterial data (33). It usually covers the bacteria that are mostly enriched in Gram-positive, Gram-negative, biofilm-forming, potentially pathogenic, mobile element-containing, aerobic, facultatively anerobic, anerobic, and oxidative stress-tolerant phenotypes (Fig. 5C). It is said that the stress caused increased gut microbial instability, gut barrier dysfunction, and systemic inflammation in geese (13, 34). Herein, we will mainly focus on stress-tolerant-related microbes. At 45 days, Lachnospirales and Oscillospirales rapidly increased, then suddenly decreased at 60 days and gradually increased at 90 days in AGF geese (Fig. S2A), which were shown to be enriched in stress-tolerant-related phenotypes.

FAPROTAX is an artificially constructed database that maps prokaryotic taxa (e.g., genus or species) to metabolic or other ecologically relevant functions (e.g., nitrification and denitrification). In the two geese groups with six samples each at 45, 60, and 90 days, we identified differences in the community and ecological functions (Fig. 5D). Based on FAPROTAX function prediction analysis, 35 functional classifications were commonly identified, such as chemoheterotrophy, fermentation, animal_parasites_or_symbionts, mammal_gut, and human_gut. To better understand the FAPROTAX database, we performed a test for differences between two functional groups at 45, 60, and 90 days (Fig. S2B). At 45, 60, and 90 days, the biological functions of chemoheterotrophy, fermentation, photoautotrophy, phototrophy, cyanobacteria, and oxygenic_photoautotrophy were significantly different in the AGF and IHF geese groups. In short, pasture supplementation can maintain the restoration of gut ecosystem function by regulating metabolic functions.

### Core microbiota decipherers’ metabolic functional features

**Fig 6:**
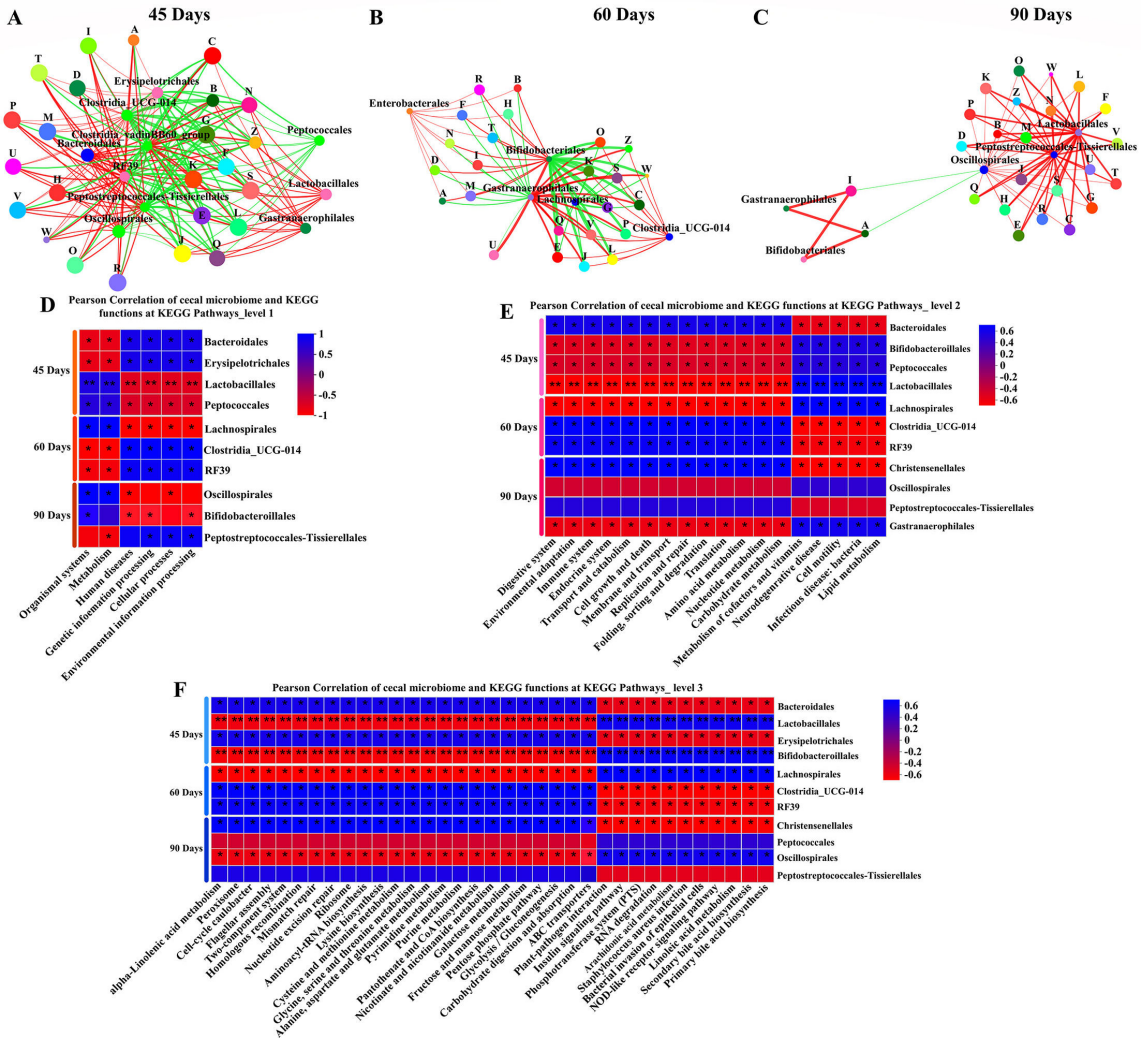
Core microbiota decipherers’ metabolic functional features. (**A–C**) Two-factor network analysis between species and microbial COG functional classifications in the 45-, 60-, and 90-day geese groups. Each co-occurring pair among host markers had an absolute Spearman’s correlation above 0.50 [the red thin line indicates a general positive correlation (0.5 < R < .70); the red thick line indicates a strong positive correlation (R > 0.70); the green thin line indicates a general negative correlation (−0.7  <  R < −0.50)] with a significance level under 0.05. Circles with distinct colors represent the species from the order level. Edge lines with distinct colors are in direct proportion to the direction of correlation (red means positive correlation; green means negative correlation). The width of the edge line represents the strength of the correlation. (**D**) Correlation heatmap chart of cecal bacterial species and microbial KEGG pathways at level 1. (**E**) Correlation heatmap chart of cecal bacterial species and microbial KEGG pathways at level 2. (**F**) Correlation heatmap chart of cecal bacterial species and microbial KEGG pathways at level 3. C, energy production and conversion; E, amino acid transport and metabolism; J, translation, ribosomal structure, and biogenesis; K, transcription; L, replication, recombination, and repair; O, posttranslational modification, protein turnover, and chaperones; P, inorganic ion transport and metabolism; Q, secondary metabolite biosynthesis, transport, and catabolism; S, function unknown; V, defense mechanisms; W, extracellular structures; Z, cytoskeleton. The results showed that the cecal bacterial community was affected by pasture intake, and there were differences in the influence of commercial diet and pasture intake on different bacterial species.

Building on the core microbiota and the key functional features found in the meat geese cecal samples, we further explored the correlation between the core microbiota and the microbial COG and KEGG pathways using Spearman’s rank correlation coefficient. As shown in Fig. 6A, *Clostridia_UCG-014* was negatively correlated with all of the metabolic functional features at 45 days of sample collection. In the 60 days of geese groups, Bifidobacteriales and Lachnospirales were negatively correlated with C, E, J, K, L, O, P, Q, S, V, W, and Z, while Gastranaerophilales was positively correlated with all of them (Fig. 6B). In the 90-d geese samples, Lactobacillales, Peptostreptococcales, and Oscillospirales were positively correlated with all of the metabolic functional features, except I and A (Fig. 6C).

For the relationship between core microbiota and the KEGG pathways, Lactobacillales, Peptococcales (45 days), Lachnospirales (60 days), Oscillospirales, and Bifidobacteriales (90 days) were significantly positively correlated with the KEGG pathways at level 1, including organismal systems, metabolism, genetic information processing, and environmental information processing (red-colored boxes) (Fig. 6D). Next, based on current functional alterations of the gut microbial KEGG pathway at level 1, we speculated to investigate the role of cecal core microbiota in modulating the other two KEGG pathways at levels 2 and 3 in geese. Significant differences between the two groups were identified in 18 pathways on KEGG level 2. Among them, 13 KEGG pathways, namely, digestive system, environmental adaptation, immune system, endocrine system, transport and catabolism, membrane transport, replication and repair, folding, sorting, and degradation, translation, amino acid metabolism, nucleotide metabolism, carbohydrate metabolism, and cell growth and death, were significantly positively correlated with Bifidobacteriales, Peptococcales, Lactobacillales, (45 days), Lachnospirales (60 days), Oscillospirales, and Gastranaerophilales (90 days) (*P* < 0.05) (red-colored boxes) (Fig. 6E). The other five KEGG pathways, namely, neurodegenerative disease, infectious disease: bacterial, cell motility, metabolism of cofactors and vitamins, and lipid metabolism were strongly negatively correlated with those of the microbiota of the 13 KEGG pathways (blue-colored boxes). Meanwhile, we also observed highly significant 35 pathways on KEGG level 3 in the IHF and AGF geese groups at 45, 60, and 90 days of sample collection. Of which, 24 pathways were strongly positively correlated with Lactobacillales, Bifidobacteroillales (45 days), Lachnospirales (60 days), Peptococcales, and Oscillospirales (90 days) (red-colored boxes) (Fig. 6F), while 11 KEGG pathways were positively correlated with Bacteroidales, Erysipelotrichales (45 days), Clostridia_UCG-014, RF39 (60 days), Christensenellales, and Peptostreptococcales-Tissierellales (90 days) (red-colored boxes) (Fig. 6F).

### Core microbiota-dependent SCFA production and their association with metabolic functional features

**Fig 7:**
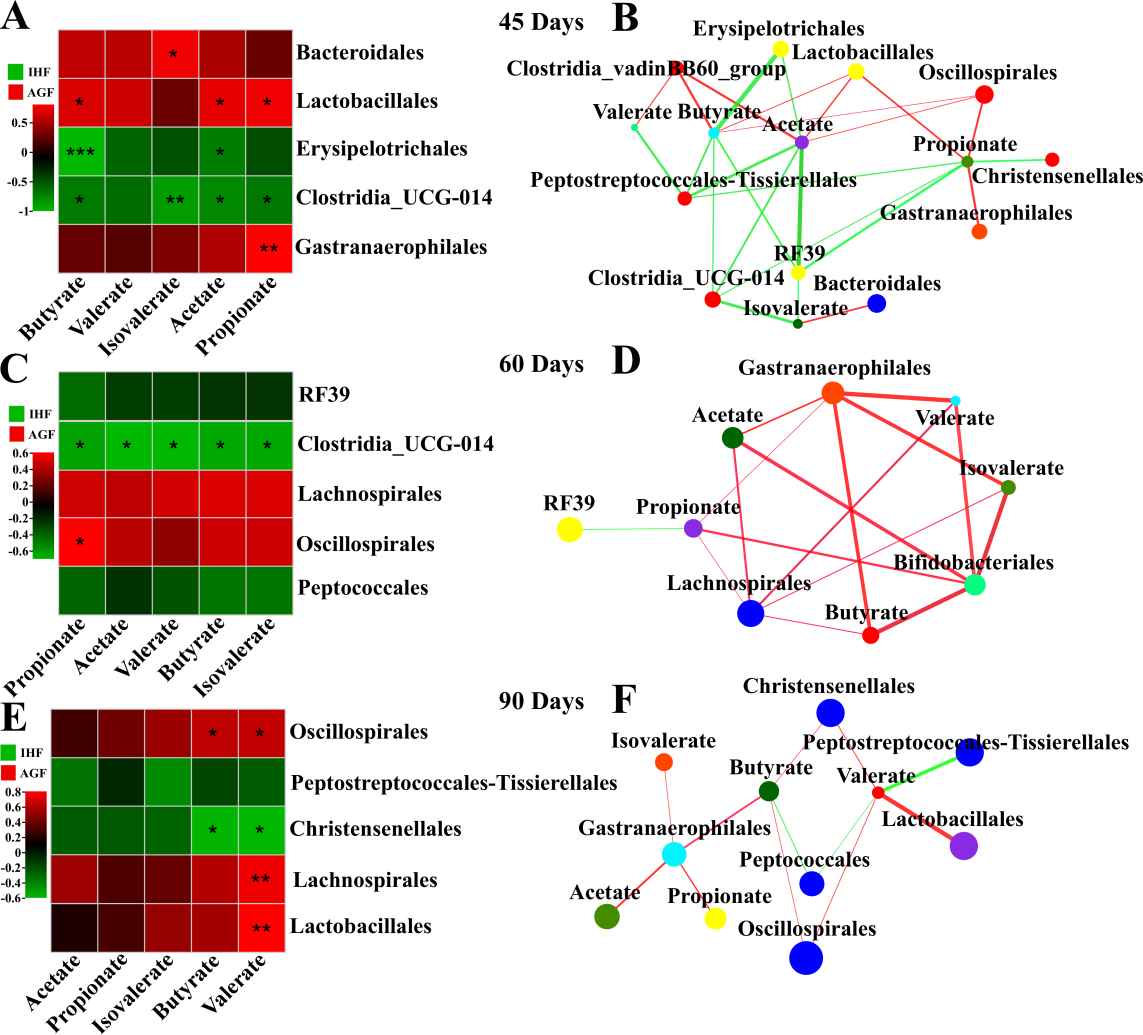
Association of SCFAs with gut microbiota composition. (A, C, and E) Correlation heatmap of SCFAs and gut microbiota. (B, D, and F) Two-factor network analysis between species at order level and SCFAs in the 45-, 60-, and 90-day geese groups. Each co-occurring pair among host markers had an absolute Spearman correlation above 0.50 [the red thin line indicates a general positive correlation (0.5 < R < .70); the red thick line indicates a strong positive correlation (R > 0.70); the green thin line indicates a general negative correlation (−0.7  <  R < −0.50)] with a significance level under 0.05. Circles with distinct colors represent the species from the order level. Edge lines with distinct colors are in direct proportion to the direction of the correlation (red means positive correlation; green means negative correlation). The width of the edge line represents the strength of the correlation.

The concentrations of SCFAs during pasture intervention differed across different phases of sample collection (Fig. 2E through I). IHF geese produced the lowest concentrations of butyrate, propionate, and valerate (Fig. 2F through H); these geese also showed the lowest response to bacterial load (Fig. 2A) due to the very low levels of dietary fibers in the commercial diet in response to cecal microbiota (Fig. 2D). We hypothesized that these key bacterial taxa may openly contribute to SCFA production and identified that the microbiota of AGF geese such as Oscillospirales and Gastranaerophilales were positively correlated with propionate, while Lactobacillales and Clostridia_vadinBB60_group were positively correlated with acetate, butyrate, and valerate in 45 days n AGF geese (Fig. 7A and B). Gastranaerophilales, Bifidobacteriales, and Lachnospirales were significantly positively correlated with acetate, butyrate, propionate, isovalerate, and valerate in 60 days of AGF geese (Fig. 7C and D). Lactobacillales, Oscillospirales, and Christensenellales were significantly positively correlated with valerate and butyrate in 90 days in AGF geese (Fig. 7E and F).

**Fig 8:**
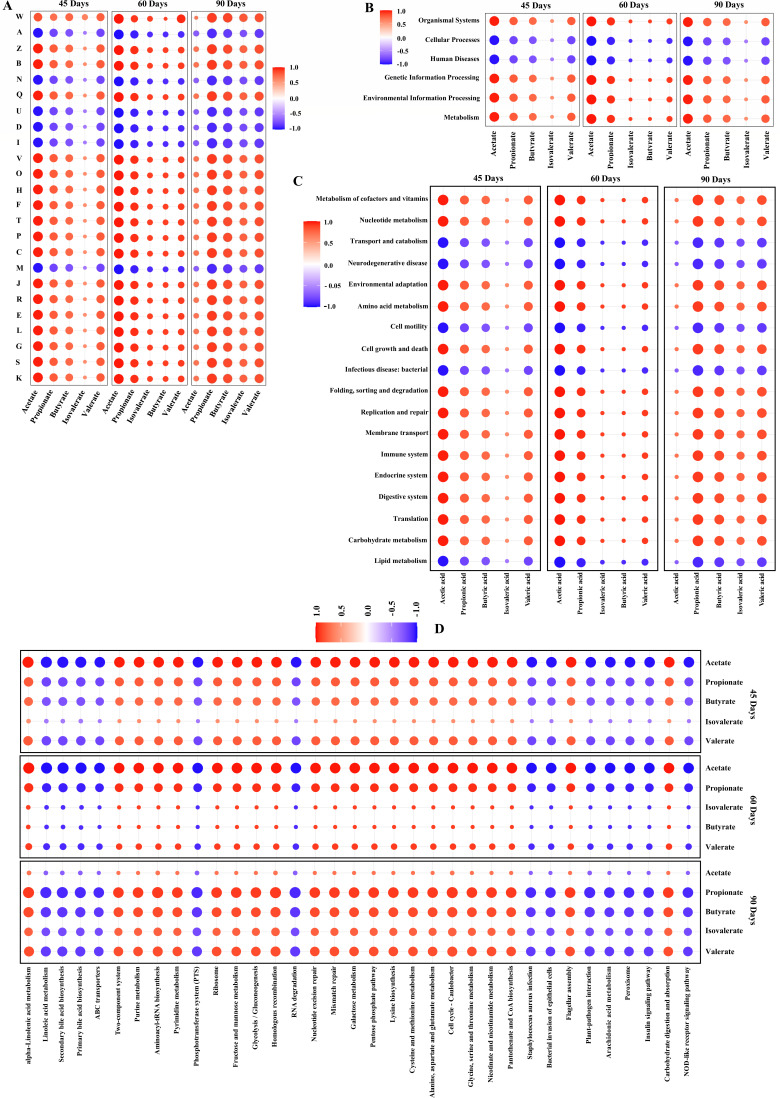
Association of SCFAs with metabolic functional features. (**A**) Correlation heatmap chart of SCFAs and COG functional classifications. A, RNA processing and modification; D, cell cycle control, cell division, and chromosome partitioning; I, lipid transport and metabolism; M, cell wall/membrane/envelope biogenesis; N, cell motility; U, intracellular trafficking, secretion, and vesicular transport. (**B**) Correlation heatmap chart of SCFAs and microbial KEGG pathways at level 1. (**C**) correlation heatmap chart of SCFAs and microbial KEGG pathways at level 2. (**D**) Correlation heatmap chart of SCFAs and microbial KEGG pathways at level 3. The results showed that the SCFAs were affected by pasture intervention, and there were differences in the influence of commercial diet and pasture intake on different COG functional classification and KEGG pathways.

Then, based on the core microbiota relationship with SCFA production, we hypothesized that these SCFAs must accelerate metabolic functional features of the gut microbiota. As shown in Fig. 8A, acetate, propionate, butyrate, and valerate, followed by isovalerate, were significantly negatively correlated with the COG functional features such as A, N, U, D, I, and M at 45, 60, and 90 days.

To further decipher the impacts of SCFAs on KEGG pathways, we correlated them by using Spearman’s rank correlation coefficient. As shown in Fig. 8B, acetate, propionate, butyrate, and valerate, followed by isovalerate, were significantly positively correlated with the KEGG pathways at level 1, including organismal systems, metabolism, genetic information processing, and environmental information processing (red-colored circles). Next, the significant differences between the two groups were identified in 18 pathways on KEGG level 2. Among them, 13 KEGG pathways, namely, the digestive system, environmental adaptation, immune system, endocrine system, transport and catabolism, membrane transport, replication and repair, folding, sorting, and degradation, translation, amino acid metabolism, nucleotide metabolism, carbohydrate metabolism, and cell growth and death, were significantly positively correlated with acetate, propionate, butyrate, and valerate, followed by isovalerate in all three phases of geese sample collection (red-colored circles) (Fig. 8C). The other five KEGG pathways, namely, neurodegenerative disease, infectious disease: bacterial, cell motility, metabolism of cofactors and vitamins, and lipid metabolism, were strongly negatively correlated with those of the SCFAs of the 13 KEGG pathways (blue-colored circles). Meanwhile, we also observed highly significant 35 pathways on KEGG level 3 in the IHF and AGF geese groups at 45, 60, and 90 days (Fig. 8D). Of which, 22 pathways were strongly positively correlated with acetate, butyrate, and isovalerate, followed by propionate and valerate (red-colored circles), while 13 KEGG pathways were negatively correlated with acetate, butyrate, isovalerate, propionate, and valerate in all phases of geese sample collection (blue-colored circles).

## DISCUSSION

Alike from ruminants, monogastric animals do not possess enzymes to degrade dietary fibers (21). In the geese, ducks, layers, and broilers, the breakdown of dietary fibers mainly depends upon colon and cecal microbial fermentation. Among them, geese are herbivores in which the crude fiber digestion is solely inhabited by cecal microbiota. It is believed that the cecal microbial activity is affected by feed (13) and age (35). In feed intervention strategies, the accumulation of green forage is believed to influence cecal microbial activity in geese (9). Therefore, we aimed that the long-term pasture supplementation from 25 days to 90 days could maintain cecal microbial composition against commercial diet-dependent cecal microbial modulations.

The main findings of our study are as follows: first, pasture intake-dependent ANOSIM-Adonis analysis (Bray–Curtis) described the significant differences between the two groups. Second, microPITA analysis to choose five criteria for microbial community selection showed that the AGF group exhibited maximum diversity, most dissimilarity, and was most representative. Third, long-term pasture supplementation was effective in maintaining gut homeostasis by balancing gut microbiota, particularly Oscillospirales, Lactobacillales, Lachnospirales, Gastranaerophilales, Bifidobacteriales, and Bacteroidales. Fifth, pasture supplementation influenced the endogenous SCFA regulation. Sixth, the higher R^2^ values for 60 days and 90 days represented the overall goodness of fit of the neutral community model. Seventh, the pasture supplementation differentiated the microbial functional features based on COG, KEGG, BugBase, and FAPROTX databases. Eighth, the positive and negative relationships of cecal microbiota and SCFAs with COG predictive functional features and KEGG pathways at levels 1, 2, and 3 describe the mechanistic role of microbiota and SCFAs in regulating energy production and conversion, amino acid transport and metabolism, lipid transport and metabolism, lysine biosynthesis, cysteine and methionine metabolism, glycine, serine, and threonine metabolism, alanine, aspartate, and glutamate metabolism, pyrimidine metabolism, purine metabolism, etc.

Few studies have reported gut microbial alterations in mice (36), geese (9, 13), broilers (22), and humans (37) after low dietary fiber intervention strategies. In the current study, commercial diet-fed geese showed low microbial composition over pasture grazing geese. According to a few studies, the lower alpha diversity has been noted to be linked with liver cirrhosis (38), obesity (39), systemic inflammation (34), and colorectal cancer (40). Considering the positive impacts of Oscillospirales in obesity-related metabolic diseases (41), its higher abundance in pasture-supplementing geese could be considered one of the next-generation probiotic candidates. Similarly, the abundance of Lactobacillales was significantly greater in AGF geese compared to those of IHF geese, which has been considered a probiotic candidate and is involved in the development of immunoregulation, microbial interactions, and anticancer activity (42). Furthermore, we observed a higher concentration of Gastranaerophilales in AGF geese compared with those of IHF geese. It is reported that the Gastranaerophilales have been observed to be involved in fermenting hemicellulose, glucose, and starch to generate butyrate in herbivores (43, 44), and Bacteroidales has been considered a carrier of ARGs, which creates a negative impact on human health (32). Surprisingly, the abundance of Bacteroidales was lowered in pasture-supplementing geese, intriguing the health-promising effect of pasture on impeding ARG production in geese.

After microbial alterations, we searched for dietary fiber degraders in geese. The main dietary fiber degraders in pasture-supplementing geese were Bacteroidales, Lactobacillales, Gastranaerophilales (45 days), Lachnospirales, and Oscillospirales (60 days and 90 days), which transform dietary fiber into SCFAs (acetate, butyrate, propionate, valerate, and isovalerate). Herein, the most notable success of our experiment was the end products of dietary fiber degraders in pasture-supplementing geese. The SCFAs are a highly precious end product of gut microbial fermentation, and it is universally acceptable that the microbiota affect host physiology through SCFA generation (21). The lower abundance of SCFAs in commercial diet-fed geese followed those studies by which the reduced abundance of SCFAs has been seen to cause obesity, IBD, and autoimmune disorders (16, 45).

At the functional level, a positive correlation was discerned between the cecal microbiota and metabolic functional features within the COG. This correlation included key categories such as energy production and conversion, amino acid transport and metabolism, lipid transport and metabolism, translation, ribosomal structure and biogenesis, as well as transcription in Wanpu geese. Noteworthily, according to previous studies, the implication of Lactobacillales and Oscillospirales was observed in diminishing the lipid transport and metabolism in geese that received pasture supplementation (46, 47). Furthermore, the gut microbiota has been acknowledged for its regulatory role in energy metabolism, as highlighted in prior research (48).

The microbiota responsible for the production of SCFAs were found to be actively participating in diverse functional categories. These encompassed energy production and conversion, amino acid transport and metabolism, translation, ribosomal structure and biogenesis, transcription, inorganic ion transport and metabolism, defense mechanisms, extracellular structures, and cytoskeleton, among others. Further analysis of predictable functional alterations in gut microbial KEGG pathways using Tax4Fun based on 16S rRNA gene sequences demonstrated that long-term dietary interventions may contribute to changes in metabolism by cecal microbiota. The altered production of SCFAs by cecal microbes may be linked to the predicted functional changes in metabolism. The generation of SCFAs is known to be regulated by interactions among host, microbiological, and environmental factors (49).

Given that the intricate interplay between SCFAs produced by cecal microbiota and the subsequent impact on metabolism, it is reasonable to hypothesize that these SCFAs might play a role in influencing immune responses, particularly in the realms of amino acid metabolism as elucidated by Wu *et al*. (50), and carbon metabolism, as outlined by Dalile *et al*. (51). The KEGG pathway predictions pertaining to the functional aspects of gut microbiota offer a valuable lens through which to comprehend the distinct expression profiles within the gut microbiota.

In our previous work, we confirmed that gut microbial lipopolysaccharide-induced ROS caused gut microbial instability, gut barrier dysfunction, and systemic inflammation in geese (13, 34). Further, to find the role of 16S microbial in phenotypic prediction, we applied the BugBase phenotypic prediction databases. Among nine phenotyping present in microbiome samples, we mainly focused on stress-tolerant-related phenotype. Intriguingly, Lachnospirales and Oscillospirales were the most abundant microbiota at the order level, which were highly enriched in stress-tolerant-related phenotype in pasture-supplementing geese. To better understand the presence of opportunistic pathogens in all analyzed cecal samples, the FAPROTAX functional prediction database was used to confirm the chemoheterotrophy and fermentation of geese in all samples from the two groups at 45, 60, and 90 days. Most of the bacteria fermenting dietary fiber in the cecum were Bacteroidales, Lactobacillales, Gastranaerophilales (45 days), Lachnospirales, and Oscillospirales (60 days and 90 days). Lactobacillales, Lachnospirales, and Oscillospirales are the potent bacteria at the order level that have been observed in fermenting dietary fibers to SCFAs (52, 53). Our results were in accordance with those of the above-mentioned studies by which the dietary fiber in the pasture was degraded by Lactobacillales, Lachnospirales, and Oscillospirales to produce acetate, butyrate, propionate, valerate, and isovalerate in geese.

Building upon the observed association between the core microbiota and SCFAs, our hypothesis posits that these SCFAs exert an influence on the metabolic functional attributes of the gut microbiota. To investigate this, we conducted a correlation analysis between SCFAs and functional features within COG and KEGG pathways at varying hierarchical levels (1, 2, and 3). At the functional level, our analysis revealed positive correlations between SCFA and COG functional features such as energy production and conversion, amino acid transport and metabolism, and translation. Conversely, negative correlations were identified with terms related to RNA processing and modification, lipid transport and metabolism, cell motility, intracellular trafficking, secretion, and vesicular transport.

Expanding our exploration to KEGG pathways, both positive and negative correlations were observed at levels 1, 2, and 3. At KEGG level 2, SCFAs demonstrated positive correlations with amino acid metabolism, carbohydrate metabolism, immune system, digestive system, and more. At level 3, specific pathways like lysine biosynthesis, cysteine and methionine metabolism, glycine, serine, and threonine metabolism, alanine, aspartate, and glutamate metabolism, pyrimidine metabolism, and purine metabolism exhibited positive correlations with SCFAs. In the context of energy metabolism in humans, certain studies have highlighted the role of SCFAs. Notably, carbon dioxide measurement from colonocytes has indicated that these cells derive a substantial portion of their energy (up to 60%–70%) through the oxidation of SCFAs, particularly butyrate, acetate, and propionate. Additionally, the liver has been identified as a key player in the processing of exogenous and endogenous acetate, serving as a substrate for long-chain fatty acids and a co-substrate for glutamine and glutamate biosynthesis (54). Further investigations have elucidated the conversion of SCFAs into acetyl-CoA, contributing to the Krebs cycle for energy generation.

The inconsistent impacts of gut microbial-induced SCFAs on lipid metabolism might contribute to the development of obesity (55). However, controversy still exists considering the role of SCFAs on lipid metabolism in geese. Strikingly, the higher production of SCFAs in pasture grazing geese was negatively correlated with the lipid metabolism pathway. Some studies regarding dietary fiber intake support our work by which SCFAs negatively correlated with lipid metabolism (26, 56). Subsequent investigations should aim to substantiate the impact of various dietary fiber sources on the regulation of functional features within SCFA-targeted COG and KEGG pathways. This verification should extend across multiple hierarchical levels (1, 2, and 3) encompassing crucial biological processes such as energy production and conversion, amino acid transport and metabolism, lipid transport and metabolism, lysine biosynthesis, cysteine and methionine metabolism, glycine, serine, and threonine metabolism, as well as alanine, aspartate, and glutamate metabolism, pyrimidine metabolism, and purine metabolism in animal systems.

In conclusion, a long-term pasture grazing system restores the cecal microbial richness against a commercial diet-fed system in geese. The major bacterial species at the order level such as Bacteroidales, Lactobacillales, Gastranaerophilales (45 days), Lachnospirales, and Oscillospirales (60 days and 90 days) were mainly influenced by the pasture grazing system. In addition, Lactobacillales, Lachnospirales, and Oscillospirales were the potent bacteria that were observed in fermenting dietary fibers to SCFAs. Finally, the involvement of core microbiota and SCFAs in differentiating COG, KEGG pathways, BugBase, and FAPROTAX functional features describes the impacts of pasture on geese’ gut health.

## MATERIALS AND METHODS

### Animals, housing, and management

Wanpu mixed-sex geese (*n* = 180, 1-day-old) were sourced from the Henan Daidai Goose Agriculture and Animal Husbandry Development Co., Ltd. (Zhumadian, China). Reaching at the age of 25 days, the geese having similar body weight were separated into two groups, i.e., (1) in-house feeding (IHF, *n* = 90) fed on commercial diet (grower diet from 25 to 45 days and a finisher diet from 46 to 90 days) (Table S4) and (2) artificial pasture grazing (ryegrass) group (AGF, *n* = 90). Geese in each group were divided into six replicates of 15 birds each with *ad libitum* supply of feed and water.

### Sample collection

At the end of 45, 60, and 90 days, six geese per group were selected for microbiome and targeted metabolomics analysis (Table S5). After 12 hours of fasting, the live weight of each geese was recorded. The geese were slaughtered by the halal method, and fresh cecal chyme was collected and kept at –80°C in liquid nitrogen.

### Determination of SCFAs

According to Liu *et al*., the concentration of SCFAs from cecal chyme samples was assessed using gas chromatography (GS) (23).

### DNA extraction and 16S rRNA gene sequencing

The microbial community genomic DNA and the bacterial 16S rRNA gene sequencing was carried out using method described by Ali *et al.* (57).

### Illumina MiSeq sequencing

The purified amplicons were combined in equimolar concentrations and subjected to paired-end sequencing (2 × 300) on an Illumina MiSeq platform (Illumina, San Diego, USA) in accordance with standard protocols provided by Majorbio Bio-Pharm Technology Co. Ltd. (Shanghai, China).

### Bioinformatics analysis of sequencing data

Paired-end reads obtained from sequencing were merged using FLASH v.1.2.11 (https://ccb.jhu.edu/software/FLASH). The raw data underwent processing and analysis using QIIME v.1.9.1 (http://qiime.org/install/index.html) to eliminate low-quality reads. Quality control of the sequences was performed using Fastp v.0.19.6 (https://github.com/OpenGene/fastp).

### Statistical analyses

### Alpha-diversity analysis

Alpha diversity metrics, specifically Shannon and Chao indices, were computed using the OTU profiles obtained from the MOTHUR program (version 1.30.2, https://mothur.org/wiki/calculators/). To assess alpha diversity among six groups, the Kruskal–Wallis H test was utilized for both Shannon and Chao indices. To account for multiple comparisons, the false discovery rate (FDR) was taken into consideration to adjust *P* values.

### Species composition analysis

Venn analysis was employed to enumerate common and unique species at the order level using R software (version 3.3.1).

### Beta-diversity analysis

The neutral community model (NCM) was established using an R script (58). Subsequently, the ANOSIM-Adonis test, implemented in R language (version 3.3.1), was employed to evaluate the statistical significance of community compositional differences among groups.

### Species difference analysis

The Wilcoxon rank-sum test, also known as the Mann–Whitney U test, was employed to analyze the significant differences between species in the two groups of samples.

### Environmental factor association analysis

Correlation heatmap diagrams were constructed by R (version 3.3.1, pheatmap package).

### Correlation with model predictive analytics

Single-factor and two-factor correlation network analyses were applied to construct a correlation network between species and environmental factors by R (plotROC package) software.

### Functional predictive analytics

To elucidate the role of the microbiota in Wanpu geese, the PICRUSt1 v.2.2.0 (phylogenetic investigation of communities by reconstruction of unobserved states) program was employed to analyze data in the context of the Cluster of Orthologous Groups (COG). Additionally, Tax4Fun v.0.3.1 (http://tax4fun.gobics.de/) was used for inferred metagenome profiling against canonical pathways of the Kyoto Encyclopedia of Genes and Genomes (KEGG). BugBase (https://bugbase.cs.umn.edu/index.html), a microbiome analysis tool, was utilized to identify high levels of phenotyping in microbiome samples for phenotypic prediction. FAPROTAX, an artificially constructed database, was applied to map prokaryotic taxa (e.g., genus or species) to metabolic or other ecologically relevant functions (e.g., nitrification and denitrification)

### MicroPITA analysis

MicroPITA (microbiomes: Picking Interesting Taxonomic Abundance) analysis was implemented using R software (version 3.3.1, Python). This analytical approach is typically employed prior to metagenomics experiments to select representative samples from a substantial pool of microbial diversity samples based on various criteria for subsequent metagenomic sequencing.

### Targeted metabolomics analysis

The data were presented as mean ± SEM. Statistical analyses were conducted using SPSS 20.0 software (IBM SPSS Statistics, Inc., 2009, Chicago, IL, USA; www.spss.com). A two-tailed unpaired Student’s *t*-test was employed for evaluating data from two groups, with significance set at *P* < 0.05. Spearman’s correlation analysis of the Euclidean distance was carried out using GraphPad Prism v. 8.3.0. For comparing host markers’ relationships, Spearman’s correlation analysis was performed using Majorbio (https://cloud.majorbio.com) and OECloud tools (https://cloud.oebiotech.cn).

## Data Availability

The unpublished microbiome data of this article are a part of such sequence data that have been uploaded in Sequence Read Achrive of NCBI under accession code: SRP395138 in our previously published article (13).

## SUPPLEMENTAL MATERIAL

Supplemental material spectrum.04188-23-s0001.docx Tables S1 to S5; Fig. S1 and S2.

ASM does not own the copyrights to Supplemental Material that may be linked to, or accessed through, an article. The authors have granted ASM a non-exclusive, world-wide license to publish the Supplemental Material files. Please contact the corresponding author directly for reuse.
