# Supplementary material for: Chronological dynamics of the gut microbiome in response to the pasture grazing system in geese
Source: Microbiol Spectr. 2024 Aug 27;12(10):e04188-23. doi: 10.1128/spectrum.04188-23 (PMC11448393; doi:10.1128/spectrum.04188-23)
Supplement: Supplemental material — Tables S1 to S5; Fig. S1 and S2. [file spectrum.04188-23-s0001.docx]

**Microbiology Spectrum**

**Chronological dynamics of gut microbiome in response to pasture grazing system in geese**

Running title: Effect of pasture grazing system on gut microbiota

Qasim Ali ^1^, Sen Ma ^1,2,3^, Umar Farooq ^4^, Boshuai Liu ^1,2,3^, Zhichang Wang ^1,2,3^, Hao Sun ^1,2,3^, Yalei Cui ^1,2,3^, Defeng Li ^1,2,3^ and Yinghua Shi ^1,2,3,*^

^1^ Department of Animal Nutrition and Feed Science, College of Animal Science and Technology, Henan Agricultural University, Zhengzhou, China

^2^ Henan Key Laboratory of Innovation and Utilization of Grassland Resources, Zhengzhou, China

^3^ Henan Herbage Engineering Technology Research Center, Zhengzhou, China

^4^ Department of Poultry Science, University of Agriculture Faisalabad, Sub Campus Toba Tek Singh, 36050, Pakistan

***Correspondence:** Yinghua Shi

Permanent address: No.15 Longzihu University Area, Zhengdong New District, Henan Agricultural University, Zhengzhou 450046, P.R. China.

**E-mail:** annysyh@henau.edu.cn

Table S1. ANOSIM-Adonis analysis (Bray-Curtis) to find the significant difference among the groups.

|  | Df | SumsOfSqs | MeanSqs | F.Model | R2 | Pr(>F) |
| --- | --- | --- | --- | --- | --- | --- |
| group_factor$G1 | 5 | 1.1379 | 0.2275 | 3.3801 | 0.3603 | 0.001 |
| Residuals | 30 | 2.0199 | 0.0673 | - | 0.6396 | - |
| Total | 35 | 3.1578 | - | - | 1 | - |

Table S2. Microbial community selection based on the microPITA program.

| Method | Sample1 | Sample2 | Sample3 | Sample4 | Sample5 | Sample6 | Sample7 | Sample8 | Sample9 | Sample10 |
| --- | --- | --- | --- | --- | --- | --- | --- | --- | --- | --- |
| Maximum diversity | AGF60_5 | IHF60_4 | AGF90_6 | AGF45_5 | AGF60_4 | AGF45_1 | IHF90_2 | AGF90_3 | AGF45_4 | AGF90_5 |
| Most dissimilar | AGF45_5 | IHF90_3 | AGF45_1 | IHF90_1 | IHF45_6 | IHF60_4 | AGF45_4 | IHF90_2 | AGF45_2 | IHF60_6 |
| Most representative | IHF90_2 | IHF45_6 | AGF90_5 | AGF60_2 | IHF45_2 | IHF60_1 | AGF60_1 | AGF45_3 | IHF45_3 | IHF60_6 |

Table S3. Core microbiota-dependent SCFAs production.

| Parameters | IHF45:AGF45 | IHF60:AGF60 | IHF90:AGF90 | SCFAs @ 45:60:90 |
| --- | --- | --- | --- | --- |
| SCFAs | 17.25:33.69 | 43.66:89.53 | 29.24:45.82 | 25.47:66.59:37.53 |
| *P* value | 0.001 | 0.0000 | 0.0000 | - |


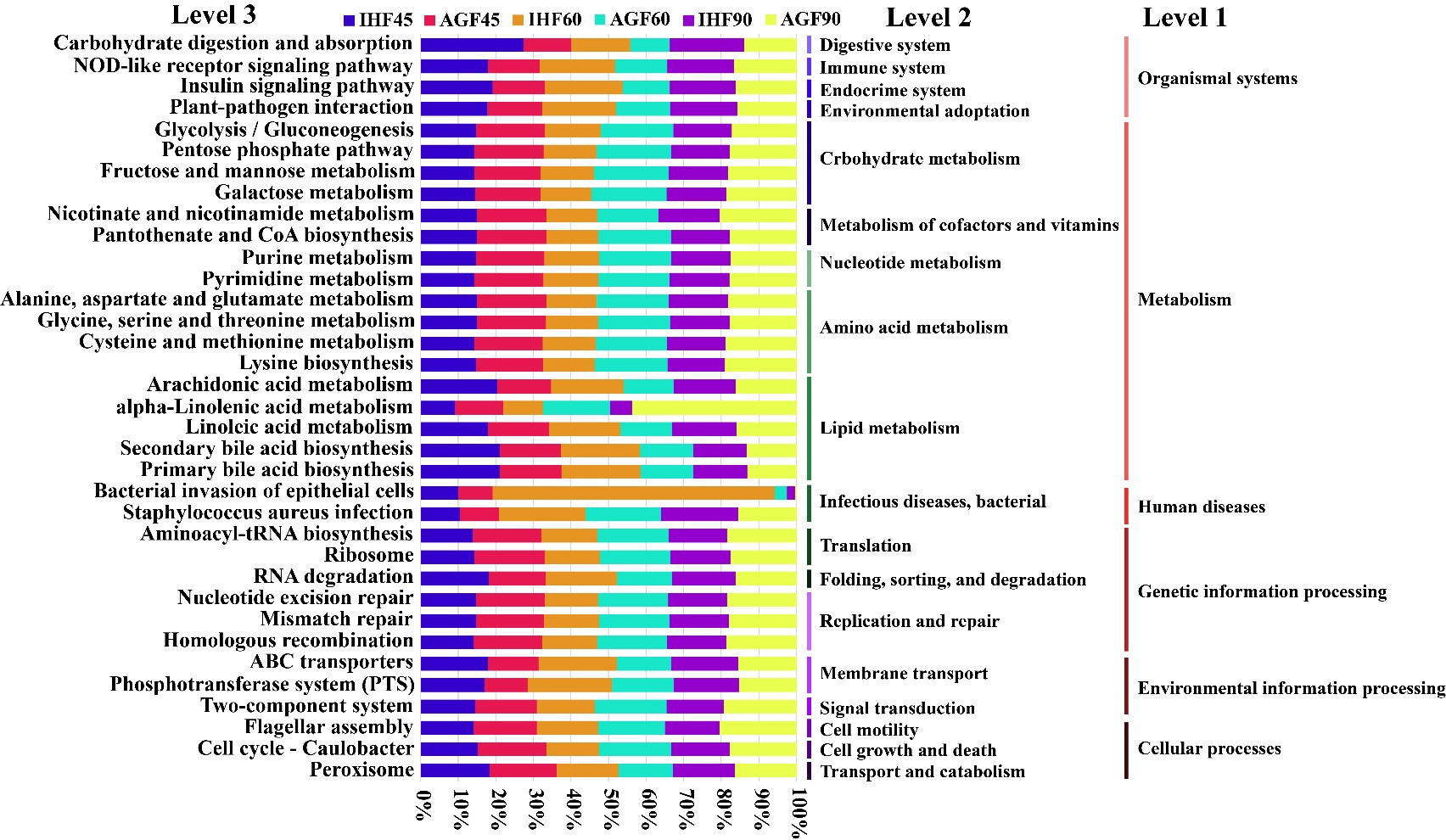
Fig. S1. Predictive functional metagenomes in response to pasture intervention. Tax4Fun was used to predict the function of potential microbial KEGG. A total of 6 KEGG pathways were predicted in cecal chyme samples at level 1, 4 in the AGF meat geese group, and 2 in the IHF meat geese group. A total of 18 KEGG pathways were predicted in cecal chyme samples at level 2, 13 in the AGF meat geese group, and 5 in the IHF meat geese group. A total of 35 KEGG pathways were predicted in cecal chyme samples at level 3, 24 in the AGF meat geese group, and 11 in the IHF meat geese group. In-house feeding system (IHF) and pasture grazing system (AGF).


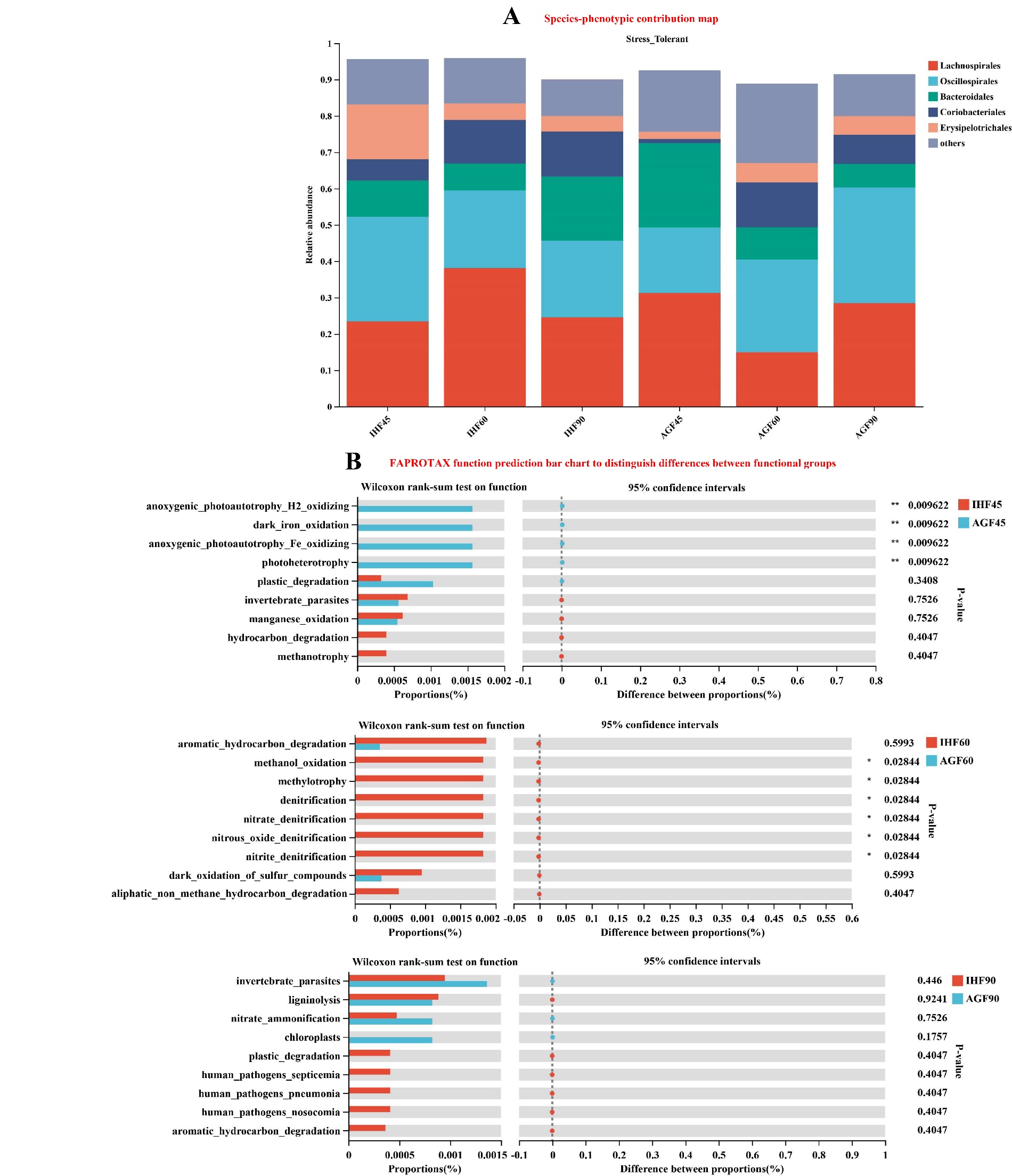


Fig. S2. Functional features of the gut microbiota isolated from Wanpu geese. (A) Species-phenotypic contribution analysis. The species-phenotypic contribution map shows the correspondence between species and phenotype. (B) FAPROTAX database to identify the results for differences between functional groups (IHF and AGF geese groups) at 45d, 60d, and 90d.

Table S4. Nutritional composition of the diet.

|  | Diets | |
| --- | --- | --- |
| Ingredients, % | Grower | Finisher |
| Wheat | 57.3 | 59 |
| Rice bran | 5 | 4 |
| Corn germ cake (exp.) | 4 | 3.2 |
| Corn oil | 5 | 7 |
| Dumpling powder | 3 | 2 |
| Corn distiller’s grains (DDGS) | 6.5 | 7 |
| Spouting germ meal | 3 | 2 |
| Soybean meal (sol.) | 7 | 6 |
| Peanut meal (sol.) | 1.5 | 1 |
| Albumen powder | 2 | 1.5 |
| Stone powder | 1.1 | 1 |
| Liquid methionine | 0.25 | 0.3 |
| MuLaoDa-2 | 1.25 | 2 |
| 201/202 gunk | 2.5 | 3 |
| Calcium hydrogen phosphate | 0.6 | 1 |
| *Chemical composition (%)* |  |  |
| Crude protein | 20.12 | 15.54 |
| Ash | 12.89 | 12.86 |
| NDF | 13.25 | 30.55 |
| ADF | 5.5 | 27.02 |
| Ca | 1.15 | 1.07 |
| P | 0.47 | 0.32 |

The nutritional composition of pasture (ryegrass) was dry matter (90%), crude protein (15.47%), ash (8%), neutral detergent fiber (65%), acid detergent fiber (38%), ether extract (3.3%), calcium (0.90%), and phosphorous (0.47%).

Table S5. Grouping of geese for transcriptomics and metabolomics analysis.

| **Group** | **Abbreviation** | **Sample name** | **Microbiome*** | **Metabolome**** |
| --- | --- | --- | --- | --- |
| In-house feeding geese | IHF | IHF1 | 1 | 1 |
| In-house feeding geese | IHF | IHF2 | 2 | 2 |
| In-house feeding geese | IHF | IHF3 | 3 | 3 |
| In-house feeding geese | IHF | IHF4 | 4 | 4 |
| In-house feeding geese | IHF | IHF5 | 5 | 5 |
| In-house feeding geese | IHF | IHF6 | 6 | 6 |
| Artificial pasture grazing geese | AGF | AGF1 | 1 | 1 |
| Artificial pasture grazing geese | AGF | AGF2 | 2 | 2 |
| Artificial pasture grazing geese | AGF | AGF3 | 3 | 3 |
| Artificial pasture grazing geese | AGF | AGF4 | 4 | 4 |
| Artificial pasture grazing geese | AGF | AGF5 | 5 | 5 |
| Artificial pasture grazing geese | AGF | AGF6 | 6 | 6 |

1) In-house feeding geese: The geese were provided only commercial feed.

2) Artificial pasture grazing geese: The geese were allowed to graze from 6:00 a.m. to 18:00 p.m. and commercial feed was provided only once a day at 19:00 p.m.

* Untargeted microbiome.

** Targeted metabolome.
